# Analysis of spike protein variants evolved in a novel *in vivo* long-term replication model for SARS-CoV-2

**DOI:** 10.3389/fcimb.2023.1280686

**Published:** 2023-11-03

**Authors:** Dongbum Kim, Jinsoo Kim, Minyoung Kim, Heedo Park, Sangkyu Park, Sony Maharjan, Kyeongbin Baek, Bo Min Kang, Suyeon Kim, Man-Seong Park, Younghee Lee, Hyung-Joo Kwon

**Affiliations:** ^1^ Institute of Medical Science, College of Medicine, Hallym University, Chuncheon, Republic of Korea; ^2^ Department of Microbiology, College of Medicine, Hallym University, Chuncheon, Republic of Korea; ^3^ Department of Microbiology, Institute for Viral Diseases, Vaccine Innovation Center, College of Medicine, Korea University, Seoul, Republic of Korea; ^4^ Department of Biochemistry, College of Natural Sciences, Chungbuk National University, Cheongju, Republic of Korea

**Keywords:** COVID-19, K18-hACE2 mouse, long-term replication model, SARS-CoV-2, variants

## Abstract

**Introduction:**

The spectrum of SARS-CoV-2 mutations have increased over time, resulting in the emergence of several variants of concern. Persistent infection is assumed to be involved in the evolution of the variants. Calu-3 human lung cancer cells persistently grow without apoptosis and release low virus titers after infection.

**Methods:**

We established a novel *in vivo* long-term replication model using xenografts of Calu-3 human lung cancer cells in immunodeficient mice. Virus replication in the tumor was monitored for 30 days and occurrence of mutations in the viral genome was determined by whole-genome deep sequencing. Viral isolates with mutations were selected after plaque forming assays and their properties were determined in cells and in K18-hACE2 mice.

**Results:**

After infection with parental SARS-CoV-2, viruses were found in the tumor tissues for up to 30 days and acquired various mutations, predominantly in the spike (S) protein, some of which increased while others fluctuated for 30 days. Three viral isolates with different combination of mutations produced higher virus titers than the parental virus in Calu-3 cells without cytopathic effects. In K18-hACE2 mice, the variants were less lethal than the parental virus. Infection with each variant induced production of cross-reactive antibodies to the receptor binding domain of parental SARS-CoV-2 S protein and provided protective immunity against subsequent challenge with parental virus.

**Discussion:**

These results suggest that most of the SARS-CoV-2 variants acquired mutations promoting host adaptation in the Calu-3 xenograft mice. This model can be used in the future to further study SARS-CoV-2 variants upon long-term replication *in vivo*.

## 1 Introduction

The RNA genome of severe acute respiratory syndrome coronavirus 2 (SARS-CoV-2) is similar to that of other SARS-related coronaviruses, encoding four major structural proteins, referred to as the spike (S), membrane (M), nucleocapsid (N), and envelope (E), and a variable number of open reading frames (ORFs) that assist in viral replication and transcription (V'kovski et al., 2021). The receptor binding domain (RBD) of the S protein facilitates viral entry into host cells via its high affinity to human angiotensin-converting enzyme 2 (ACE2) (Shang et al., 2020). SARS-CoV and Middle East respiratory syndrome coronavirus (MERS-CoV) caused relatively small epidemic outbreaks with high pathogenicity and mortality in 2002 and 2012, respectively (Hu et al., 2021). Although the item caused by SARS-CoV and MERS-CoV each lasted less than a year, the COVID-19 pandemic has lasted over 3 years, raising fundamental issues regarding the frequent mutation and evolutionary patterns of viral genes (Chavda et al., 2022). Specifically, increased mutation seems to influence virus transmissibility (Volz et al., 2021; Zhou et al., 2021; Carabelli et al., 2023), pathogenicity(Volz et al., 2021), infectivity (Zhang et al., 2020; Wu et al., 2021), immune escape (Thorne et al., 2022; Carabelli et al., 2023), and drug resistance (Alteri et al., 2022; Donovan-Banfield et al., 2022).

Shortly after the COVID-19 outbreak, a SARS-CoV-2 variant harboring a D614G mutation in the S protein rapidly transmit as a dominant strain, and more variants have since emerged (Volz et al., 2021; Zhou et al., 2021). Successively emerging strains of SARS-CoV-2 have been labeled as Alpha, Beta, Gamma, Delta, Epsilon, Eta, Iota, Kappa, Mu, Zeta, and Omicron variants. Omicron is currently the predominant variant worldwide, accounting for > 99% of the variant genome frequency. Compared with the previous variants, the Omicron variant contains 30 or more mutations in the S protein (Zhou et al., 2023).

Individuals that are immunocompromised because of cancer, chemotherapy, organ transplantation, HIV infection, chronic diabetes, or other reasons cannot neutralize SARS-CoV-2, so they sustain persistent infection for long periods (e.g., over 3 months in a patient with chronic lymphoid leukemia) (Avanzato et al., 2020; Borges et al., 2021; Clark et al., 2021; Kemp et al., 2021). When viruses multiply inside human tissues for long periods, they can evolve adaptations to evade host immune responses and resist the effects of antiviral drugs, increasing the probability that a novel strain will emerge. Hence, immunocompromised individuals represent a likely source of new SARS-CoV-2 variants (Avanzato et al., 2020; Clark et al., 2021).

According to the previous investigation by others, several mutations reported in natural variants were recapitulated by serial passaging in human cell lines, with some differences between colon epithelial cells and lung epithelial cells (Chung et al., 2022). Some mutations involved in SARS-CoV-2 entry into cells were evolved by serial passage in human cell lines without ACE2 or cellular proteases (Puray-Chavez et al., 2021; Chaudhry et al., 2022). These results suggest the specific host cell environment can affect the selection of mutations. In this context, we aimed to establish an *in vivo* long-term replication model. Previously, we reported that the response of host cells after SARS-CoV-2 infection, especially apoptosis, differ depending on the cell type (Park et al., 2021a). Vero monkey kidney cells show high virus production and an apoptotic phenotype after infection, whereas Calu-3 human lung cancer cells persistently grow without apoptosis and release low virus titers after infection (Park et al., 2021a) probably because of interferon signaling (Young et al., 2003). Therefore, we selected Calu-3 cells as a host cell, created xenograft tumors derived from Calu-3 cells in severely immune deficient mice, and infected the tumors with parental SARS-CoV-2 to establish a long-lived *in vivo* incubator for SARS-CoV-2. Here, we found that new SARS-CoV-2 variants emerged in the tumor tissues, which we characterized *in vitro* and *in vivo.* Our results suggest that the immunodeficient mouse xenograft model can be useful for investigations of SARS-CoV-2 variants evolved from long-term replication *in vivo*.

## 2 Materials and methods

### 2.1 Cell culture and virus

Human airway epithelial Calu-3 cells (Catalog No. 30055) and African green monkey kidney Vero E6 cells (Catalog No. 21587) were obtained from the Korean Cell Line Bank (Seoul, Korea). Every cell was maintained in Dulbecco’s modified Eagle’s medium (DMEM; Thermo Fisher Scientific, Waltham, MA, USA) containing 10% fetal bovine serum (FBS; Thermo Fisher Scientific), 25 mM HEPES, 100 U/mL penicillin, and 100 μg/mL streptomycin at 37°C in a 5% CO_2_ incubator. Parental SARS-CoV-2 (hCoV-19/Korea/KCDC03/2020, lineage A, NCCP43326, GenBank accession no. MW466791.1) was provided by the National Culture Collection for Pathogens of Korea (Osong, Korea).

### 2.2 Virus amplification and determination of virus titers by plaque assay

SARS-CoV-2 amplification was performed in Vero E6 cells as described previously (Park et al., 2021a; Kim et al., 2021b). Vero E6 cells (7 × 10^5^ cells/well) were cultured overnight on six-well plates (Corning, NY, USA) in DMEM containing 10% FBS. The cells were then infected with SARS-CoV-2 at a multiplicity of infection (MOI) of 0.01 for 1 h at 37°C with shaking every 20 min in a CO_2_ incubator. DMEM containing 2% FBS was then added, the cells were cultured for 72 h, and the culture supernatants were collected by centrifugation. Virus titers (pfu, plaque forming unit) in the culture supernatants and homogenates of tumor tissues were quantified by plaque assay on Vero E6 cells as described previously (Park et al., 2021b; Kim et al., 2022a), Virus stocks (1 × 10^7^ pfu/mL) were stored at -70°C. SARS-CoV-2 amplification and cell culture procedures were performed in biosafety level 3 (BSL-3) conditions at the Research Institute of Medical-Bio Convergence of Hallym University with approval of the Institutional Biosafety Committee (IBC) of Hallym University (Hallym2020-12, Hallym2021-04, Hallym2022-03).

### 2.3 Cell viability assay

Vero E6 cells (5 × 10^3^ cells/well) or Calu-3 cells (5 × 10^3^ cells/well) were cultured on 96-well plates for 18 h. The cells were then washed with PBS and infected with SARS-CoV-2 in PBS at an MOI of 0.01, 0.05, or 0.1 for 1 h in a 5% CO_2_ incubator at 37°C with shaking every 20 min. The cells were then washed with PBS, and DMEM containing 2% FBS was added to each well. After 72 h, the viability of SARS-CoV-2–infected cells was assessed by cell-counting kit-8 (CCK-8) assay (Catalog No. CK04. Dojindo Molecular Technologies, Kumamoto, Japan) measuring the cellular dehydrogenase activity. After adding CCK-8 reagent, cell viability was determined by measuring the absorbance at 450 nm following the manufacturer’s instructions.

### 2.4 Animals

For the mouse xenograft model, four-week-old female NOD/ShiLtJ-Rag2*^em1AMC^* Il2rg*^em1AMC^* (NRGA) mice were purchased from JA BIO, Inc. (Suwon, Korea). For the virus challenge experiments, seven-week-old male K18-hACE2 transgenic mice (B6.Cg-Tg(K18-ACE2)2Prlmn/J) were purchased from the Jackson Laboratory (Stock. No. 034860, Bar Harbor, ME, USA). Four-week-old female C57BL/6J mice were purchased from Nara Biotech, Inc. (Seoul, Korea). All mice were maintained under specific pathogen-free conditions in a controlled environment (20–25°C, 40–45% humidity, 12-h light/dark cycle, *ad libitum* access to food and water) in the Experimental Animal Center of Hallym University. Humane endpoints were planned whereby the mice would be anesthetized by intraperitoneal injection of 0.2 mL avertin (Sigma-Aldrich, St. Louis, MO, USA) and euthanized by cervical dislocation in accordance with the approved Institutional Animal Care and Use Committee (IACUC) protocol if they lost 30% of adult body weight, reached a tumor volume of 1,000 mm^3^, or exhibited evidence of debilitation, pain, or distress such as hunched posture, rough haircoat, reduced food consumption, emaciation, inactivity, difficulty ambulating, or respiratory problems. No live mice reached the humane endpoint criteria during the experiments. All animal procedures were approved by the IACUC of Hallym University (Hallym2020-26, Hallym2021-73). Animal experiments involving SARS-CoV-2 infection were performed in animal biosafety level 3 (ABSL-3) conditions in the Research Institute of Medical-Bio Convergence of Hallym University in accordance with the recommendation of the IBC of Hallym University (Hallym2020-12, Hallym2021-04, Hallym2022-03).

### 2.5 Mouse xenograft model and virus infection experiments

Calu-3 cells (2 × 10^6^ cells/mouse) in 50% Matrigel (Corning, PBS/Matrigel, 1:1 *v*/*v*) were subcutaneously inoculated into the right flank of four-week-old female NRGA mice. After tumor volumes reached an average of 100 mm^3^, the mice (n = 3/group or n = 6/group) were intratumorally infected with 1 × 10^6^ pfu of parental SARS-CoV-2. Then, the mice were observed daily for body weight, clinical signs, and survival. Tumor diameters were estimated with calipers. The tumor volumes were measured as width^2^ × length/2 as described previously (Wu et al., 2018). The infected mice were sacrificed 3, 15, or 30 days post-infection (dpi), and the tumors were surgically excised and weighed. To measure the virus distribution in the infected mice, tumors, lungs, brains, and blood were collected at 30 dpi. Each organ was homogenized with Tissue Lyser II (Qiagen, Germantown, MD, USA), and then collected supernatants from the homogenates by centrifugation. The virus titer of each organ was determined from the supernatants of the homogenates by plaque assay and quantitative real-time reverse-transcription PCR (qRT-PCR).

### 2.6 Virus infection in C57BL/6J mice

Mice (n=3) were inoculated intraperitoneally with live parental SARS-CoV-2 at a dose of 1×10^6^ pfu per mouse. At 14 dpi, mouse sera were obtained by orbital bleeding to estimate the production of SARS-CoV-2 S protein-specific antibody.

### 2.7 Western blot

SARS-CoV-2–infected tumors were homogenized and lysed in cell lysis buffer (10 mM HEPES, 150 mM NaCl, 5 mM EDTA, 100 mM NaF, 2 mM Na_3_VO_4_, protease inhibitor cocktail, and 1% NP-40). Cell lysates were collected by centrifugation. The cell lysates were separated on a 4–12% Bis-Tris gradient gel (Thermo Fisher Scientific) for western blot analysis with anti-SARS-CoV-2 N protein monoclonal antibody (0.5 μg/mL, anti-SARS-CoV-2 N mAb, clone 1G10C4 mAb) (Kim et al., 2021a) and anti-β-actin antibody (Catalogue No. A5316 (1:5000), Sigma-Aldrich). A horseradish peroxidase (HRP)-conjugated donkey anti-mouse IgG (H+L) polyclonal antibody (Catalogue No. 715-035-150 (1:5000), Jackson ImmunoResearch Laboratories, Inc., West Grove, PA, USA) was used as secondary antibody. Immunoreactive bands were measured with an enhanced chemiluminescence (ECL) solution (Thermo Fisher Scientific).

### 2.8 qRT-PCR

SARS-CoV-2-infected tumors were homogenized with Tissue Lyser II, and the supernatants were collected by centrifugation. Total RNAs were extracted from the supernatants with TRIzol Reagent (Invitrogen, Carlsbad, CA, USA) and a PureLink™ RNA Mini Kit (Invitrogen). Then, cDNA (50 μL) was synthesized using a Reverse Transcription System Kit (Promega, Madison, WI, USA). Expression of the RNA-dependent RNA polymerase (*RdRP*) gene of parental SARS-CoV-2 was quantified using the following primers as previously described (Park et al., 2021a; Park et al., 2021b): forward primer, 5′-GTGAAATGGTCATGTGTGGCGG-3’; reverse primer, 5’-CAAATGTTAAAAACACTATTAGCATA-3’; TaqMan probe, 5’-FAM-CAGGTGGAACCTCATCAGGAGATGC-TAMRA-3’. Primers and the probe were synthesized by Genotech. 10 µL of GoTaq Probe qPCR Master Mix (Promega) was added to 10 µL of the reaction mixture containing 250 nM each of the forward and reverse primers, 125 nM probe, and 1 μL of cDNA. After denaturation at 95°C for 5 min, 45 cycles at 95°C for 15 sec and 60°C for 1 min were performed in the Rotor-Gene Q real-time PCR cycler (Qiagen). The numbers of copies of the *RdRP* gene in the samples were calculated using an *RdRP* cDNA standard curve.

### 2.9 Hematoxylin and eosin staining

Tissue sections were investigated for histopathologic analysis as previously described (Baek et al., 2022). In brief, tumor tissues were prepared at 3, 15, 30 dpi and stained with Gill’s Hematoxylin V (Muto Pure Chemicals, Tokyo, Japan), and counterstained with Eosin Y solution (Abcam, Cambridge, UK). The H&E-stained sections were observed with an Eclipse E200 microscope (Nikon, Tokyo, Japan).

### 2.10 Immunohistochemistry

Tissue sections were rehydrated and antigen retrieval was performed in citrate buffer (pH 6.0: ScyTek Laboratories). Each slide was reacted with biotinylated anti-SARS-CoV-2 N monoclonal antibody (1G10C4 mAb, 1 μg/slide) (Kim et al., 2022b), rabbit anti-SARS-CoV-2 S polyclonal antibody (Catalogue No. 40591-T62, 1 μg/slide, Sino Biological, Houston, TX, USA), or rabbit anti-ACE2 polyclonal antibody (Catalogue No. MBS4750512 (1:200), MyBioSource, San Diego, CA, USA). The slides stained with rabbit anti-SARS-CoV-2 S polyclonal antibody or rabbit anti-ACE2 antibody were further incubated with biotinylated horse anti-rabbit antibody (Catalogue No. BP 1100, Vector Laboratories, Burlingame, CA, USA). All slides were then reacted with HRP-conjugated streptavidin (Catalogue No. PK-6100 (1:200), Vector Laboratories). The HRP reaction was measured with 3, 3’-diaminobenzidine (DAB, Thermo Fisher Scientific). The slides were counterstained with Gill’s Hematoxylin V. The H&E-stained sections were observed with an Eclipse E200 microscope.

### 2.11 Viral RNA extraction from tumor tissues and whole-genome deep sequencing analysis

Total RNA samples were extracted from the supernatants of virus-infected tumor homogenates and sent to Macrogen, Inc. (Seoul, Korea) for cDNA library construction and whole-genome deep sequencing. The libraries were quantified using Kapa Library Quantification kits for Illumina Sequencing platforms (Kapa Biosystems, Wilmington, MA, USA). Sequencing was performed using an Illumina NovaSeq system (Illumina, Inc., San Diego, CA, USA). The sequencing data were filtered with FastQC (v0.11.8) (https://www.bioinformatics.babraham.ac.uk/projects/fastqc/) and Trimmomatic (v0.38) (Bolger et al., 2014) to check the read quality and reduce biases for analysis. Filtered data were mapped to reference sequences using BWA (v0.7.17) (Li and Durbin, 2010) with the MEM algorithm and modified with Sambamba (v0.6.7) (Tarasov et al., 2015). SAMtools (v.1.6) (Li, 2011) and BCFtools (v.1.6) (http://samtools.github.io/bcftools/call-m.pdf) were used for genome coverage determination, mapping ratio calculation, and variant calling. At this step, single-nucleotide polymorphisms and short insertion or deletion candidates with Phred score over 30 (base call accuracy of 99.9%) were captured and annotated with SnpEff (v.4.3t) (Cingolani et al., 2012) to predict the effects of genetic variants. The sequences of parental SARS-CoV-2 (hCoV-19/Korea/KCDC03/2020, lineage A, NCCP43326/2020, GenBank accession no. MW466791.1.) were used as a reference. The accession numbers for the SARS-CoV-2 parental proteins are listed in **Supplementary Table 1**.

### 2.12 Analysis of the S protein sequences of the variants by reverse-transcription PCR

SARS-CoV-2-infected tumor tissues were homogenized, and the supernatants were collected. Total RNAs were extracted from the supernatants of the homogenates, and cDNA was generated. For cloning of the S protein N-terminal region including the RBD (2726 bp, from -46 to 2680), standard PCR was performed for 25 cycles using AccuPrime™ Taq DNA polymerase (Invitrogen) and the following primer sets with Not I and Kpn I restriction sites included at the 5′ and 3′ ends, respectively: CoV2-S-5’ primer, 5’-TATAGCGGCCGCCAGAGTTGTTATTTCTAGTGATGTTC-3’; CoV2-S-mid-3’ primer, 5’-TATAGGTACCATGCAGCACCTGCACCAAAG-3’. The PCR products were inserted into a modified pcDNA 3.4 expression vector (Thermo Fisher Scientific), and the nucleotide sequences were verified by direct DNA sequencing using the following primers: S1-5’p forward primer, 5’-CTGGTGATTCTTCTTCAGGT-3’; S2-5’p forward primer, 5’-GAACTTCTACATGCACCAGC-3’; S0-3’p reverse primer, 5’-GTGCACAGTCTACAGCATCT-3’; pcDNA3.4 reverse primer, 5’-CAACATAGTTAAGAATACCAGTC-3’.

### 2.13 Isolation of viral plaques and sequence analysis of viral isolates

SARS-CoV-2-infected tumors were homogenized, and the supernatants were collected. For viral plaque isolation, serially diluted supernatants were supplemented onto Vero E6 cells (7 × 10^5^ cells/well) on six-well plates. After 1 h of infection, 3 mL/well DMEM/F12 medium (Thermo Fisher Scientific) containing 2% bacteriological agar (Oxoid™, Thermo Fisher Scientific) was added. After incubation at 37°C for 3 days, five plaques from each homogenate of three tumors were collected, and each isolate was amplified in Vero E6 cells. The S protein sequences of the isolates were analyzed by reverse-transcription PCR and direct DNA sequencing, and three variants (S-1, S-6, and S-9) were selected and analyzed further by whole-genome deep sequencing. The virus stocks of the variants were stored at -70°C.

### 2.14 Virus challenge experiments

Eight-week-old male hemizygous K18-hACE2 transgenic mice (B6.Cg-Tg(K18-ACE2)2Prlmn/J) were anesthetized by exposure to 1–2% isoflurane (Hana Pharm. Co. Ltd., Seoul, Korea) and then intranasally inoculated with parental SARS-CoV-2 or variants as previously described (Kim et al., 2023). To investigate the LD_50_ for each virus, mice (n = 5/group) were intranasally inoculated with serial dilutions of the viruses and monitored daily for clinical signs, body weight, and survival for up to 15 days. The LD_50_ was defined as the pfu of the virus that generated lethality in 50% of the mice. LD_50_ titers were estimated by the method of Reed and Muench (Reed and Muench, 1938). At 15 dpi with 3 × 10^4^ pfu of parental SARS-CoV-2 or variants (n = 10/group), live mice were intranasally challenged with 3 × 10^4^ pfu of parental SARS-CoV-2 and then monitored for another 15 days for clinical signs and body weight. To investigate virus-specific antibody production and neutralizing antibody titers, blood samples were collected by retro-orbital bleeding 14 days after the initial intranasal inoculation and by cardiac puncture 15 days after the subsequent intranasal challenge. Serum samples were prepared and stored at -80°C. To examine the viral loads after inoculation with parental SARS-CoV-2 or variants, mice (n = 8/group) were sacrificed 5 days after intranasal inoculation, and turbinates and lungs were collected for analysis. The turbinates and lungs were weighed and homogenized, and virus titers were estimated by plaque assay.

### 2.15 RBD-specific Ig ELISA

Ninety-six–well immunoplates (Nunc, Roskilde, Denmark) were coated with recombinant RBD of parental SARS-CoV-2 S protein (1 μg/mL, Catalog No. SPD-C82E9, Acrobiosystems, Newark, DE, USA) in carbonate buffer (pH 9.6) and incubated overnight at 4°C. The coated plates were blocked with PBST containing 1% BSA and then washed three times with PBST. Twofold serially diluted sera were then transferred to each well and incubated for 2 h at room temperature. To calculate IgG amounts, serially diluted normal mouse IgG was coated in the plate for the standard curve. Then, the plates were washed and incubated with HRP-conjugated goat anti-mouse IgG antibody (Catalog No.1030-05 (1:200), Southern Biotechnology Associates, Birmingham, AL, USA) for 1 h. The plates were washed with PBST, and the HRP activity was measured by colorimetric reaction with the substrate 3,3’,5,5’-tetramethylbenzidine (TMB; Kirkegaard and Perry Laboratories, Gaithersburg, MD, USA). The absorbance was estimated at 450 nm using a Spectra Max 250 microplate reader (Molecular Devices, San Jose, CA, USA).

### 2.16 Plaque reduction neutralization test

Vero E6 cells (7 × 10^5^ cells/well) were cultured on six-well plates for 18 h. SARS-CoV-2 wild type was pre-incubated with twofold serially diluted mouse serum for 1 h at 37°C. After the Vero E6 cells were washed with PBS, the virus-serum mixture was added. After the cells were infected for 1 h with shaking every 20 min, the supernatant was removed and 3 ml of DMEM/F12 medium (Thermo Fisher Scientific) containing 0.6% bacteriological agar was added. The plates were cultured for 72 h and then stained with 0.1% crystal violet to visualize plaque formation. Antibody titers were calculated using the Spearman-Kärber method (Hamilton and Russo, 1977) and percentage of inhibition ≥50% (PRNT_50_) is considered a positive cutoff for seroconversion against SARS-CoV-2.

### 2.17 Surrogate SARS-CoV-2 virus neutralization test

Recombinant parental SARS-CoV-2 S protein RBD-HRP fusion protein (RBD-HRP protein, Catalogue No. Z03594) and hACE2 protein (Catalogue No. Z03516) were purchased from GenScript (Piscataway, NJ, USA). The sVNT was performed as previously described (Kim et al., 2022c). Briefly, hACE2 protein (1 µg/mL) was coated on a 96-well plate and then blocked with PBS containing 1% BSA. Then, 50 µL RBD-HRP protein (2 µg/mL) in PBST and 50 µL twofold serially diluted serum were mixed and incubated for 30 min. One hundred microliters of the mixture were then added to the hACE2-coated 96-well plate and incubated for 15 min. The plate was then washed with PBST. The HRP activity was measured by colorimetric reaction with the substrate TMB, and absorbance was measured at 450 nm. The absorbance values were normalized with the values of PBS (negative control) and RBD-HRP protein alone (positive control) in hACE2-coated wells (Perera et al., 2020). The surrogate virus neutralization titer (sVNT_50_) of the serum was defined as the reciprocal value of the sample dilution that showed a 50% reduction of signal at 450 nm.

### 2.18 Statistical analysis

Results are shown as the mean ± standard error of the mean (SEM). Differences between the samples were analyzed using a two-sided unpaired Student’s *t*-test (Instat; GraphPad Inc.). *p*-values<0.05 were considered statistically significant.

## 3 Results

### 3.1 Establishment of a long-term replication model using lung cancer xenografts in mice

**Figure 1:**
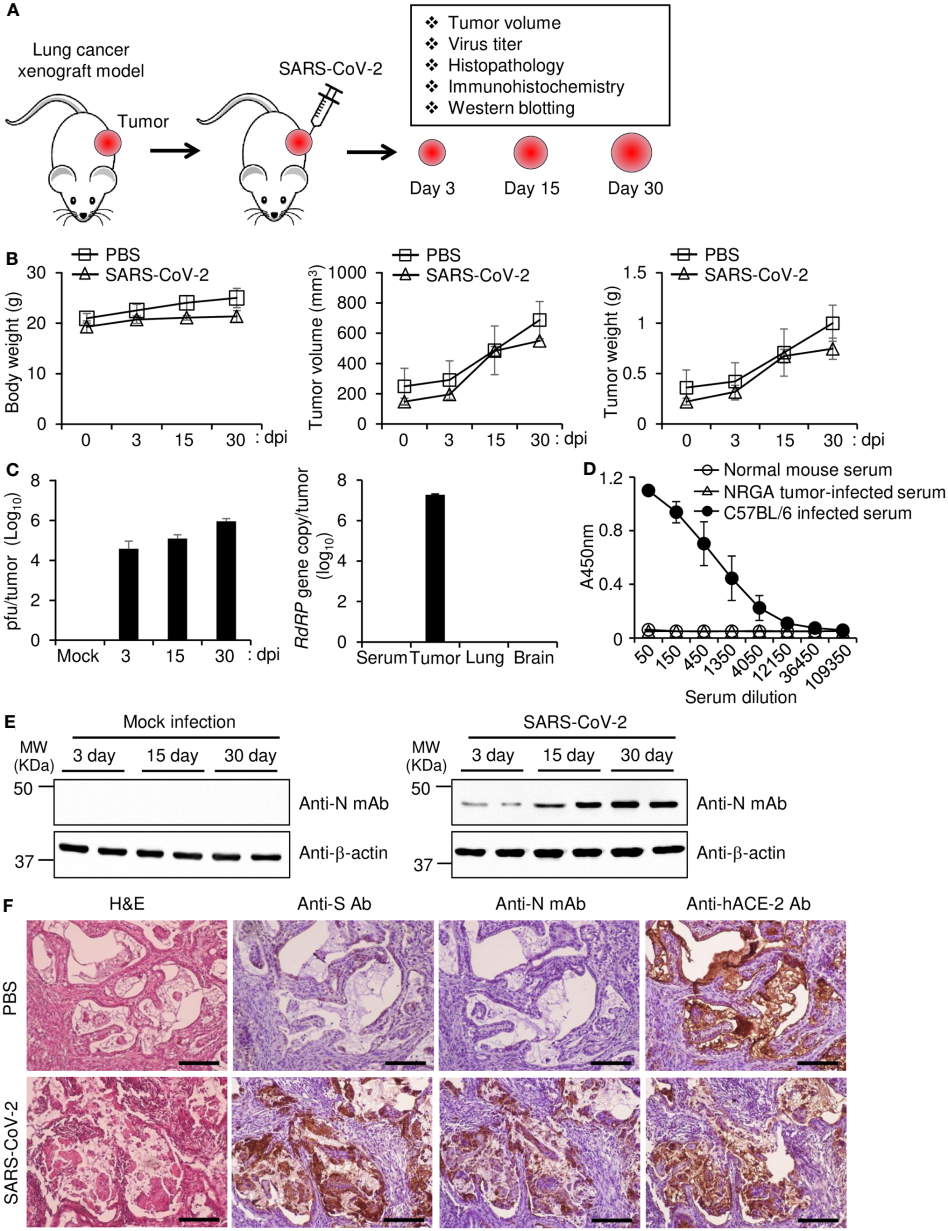
Replication of SARS-CoV-2 in a lung tumor xenograft mouse model. **(A)** Schematic diagram of the experimental procedure. Calu-3 cells were subcutaneously implanted into the right flank of NRGA mice (n = 3 per group). After the tumor volume reached 100 mm^3^, the tumors were inoculated with 1 × 10^6^ pfu of parental SARS-CoV-2, and the virus titers in the tumors were analyzed at 3, 15, and 30 dpi. **(B)** Body weight of each infection group (left panel). Tumor volume of each infection group (middle panel). Tumor weight of each infection group (right panel). **(C)** Replication of SARS-CoV-2 in lung tumors. Tumor tissues were collected at 3, 15, and 30 dpi. Viral titers in the supernatants of tumor homogenates were determined by plaque assay (left panel). Serum, tumor, lung, and brain were collected at 30 dpi, and viral titers were determined by qRT-PCR analysis for the SARS-CoV-2 *RdRP* gene (right panel). **(D)** Sera were collected at 30 dpi from NRGA mice (n=3) intratumorally infected with 1 × 10^6^ pfu of parental SARS-CoV-2 and at 14 dpi from C57BL/6J (n=3) intraperitoneally infected with 1 × 10^6^ pfu of parental SARS-CoV-2. The parental SARS-CoV-2 S protein RBD-specific IgG titers in the sera were determined by ELISA. **(E)** Expression of SARS-CoV-2 N protein in tumor tissues. The expression levels of SARS-CoV-2 N protein in mock-infected and SARS-CoV-2-infected tumor homogenates were determined by western blot. Expression of β-actin was used as a control. **(F)** Expression of SARS-CoV-2 N protein and S protein in tumor tissues. Mice (n=2) were sacrificed at 30 dpi. Paraformaldehyde-fixed, paraffin-embedded tumor tissues were sliced to 5 µm thickness, and H&E staining and immunohistochemical staining were performed to detect SARS-CoV-2 S protein and N protein and hACE2. Scale bars, 25 µm. dpi, days post-infection; Anti-N mAb, anti-SARS-CoV-2 N protein monoclonal antibody; Anti-S Ab, anti-SARS-CoV-2 S protein polyclonal antibody.

To test our hypothesis that Calu-3 cells can be used in mice to provide long-term replication model for SARS-CoV-2, we first induced xenograft tumors into genetically immunodeficient NOD/ShiLtJ-Rag2*^em1AMC^* Il2rg*^em1AMC^* (NRGA) mice using Calu-3 cells. We then infected the xenograft tumors with parental SARS-CoV-2 and monitored the mice at 3, 15, and 30 dpi as depicted in **Figure 1A**. The infected mice showed similar body weight, tumor volume, and tumor weight compared to uninfected controls, suggesting that virus infection did not induce prominent side effects (**Figure 1B**). Importantly, at 30 dpi, approximately 10^6^ pfu of virus were found in the tumors but not the sera, lungs, or brains of the mice (**Figure 1C**). Whereas C57BL/6J mice intraperitoneally infected with parental SARS-CoV-2 produced S protein-specific antibody, the NRGA mice did not produce antibodies specific to S protein of parental SARS-CoV-2 as we expected (**Figure 1D**). Therefore, we confirmed that there is no selective pressure against virus in our model in terms of immunological aspect. As shown in **Figure 1E**, expression of the viral N protein increased during the course of infection, suggesting persistent and increasing virus replication in the tumors. Immunohistochemistry data revealed persistent expression of the viral S and N proteins along with hACE2 in the tumor tissues (**Figure 1F**). Taken together, our results confirmed that the xenograft model established with highly permissible Calu-3 lung cancer cells can be used as a model for long-term SARS-CoV-2 replication without harmful effects on infected mice up to one month.

### 3.2 SARS-CoV-2 mutations in cell culture during serial passage

**Figure 2:**
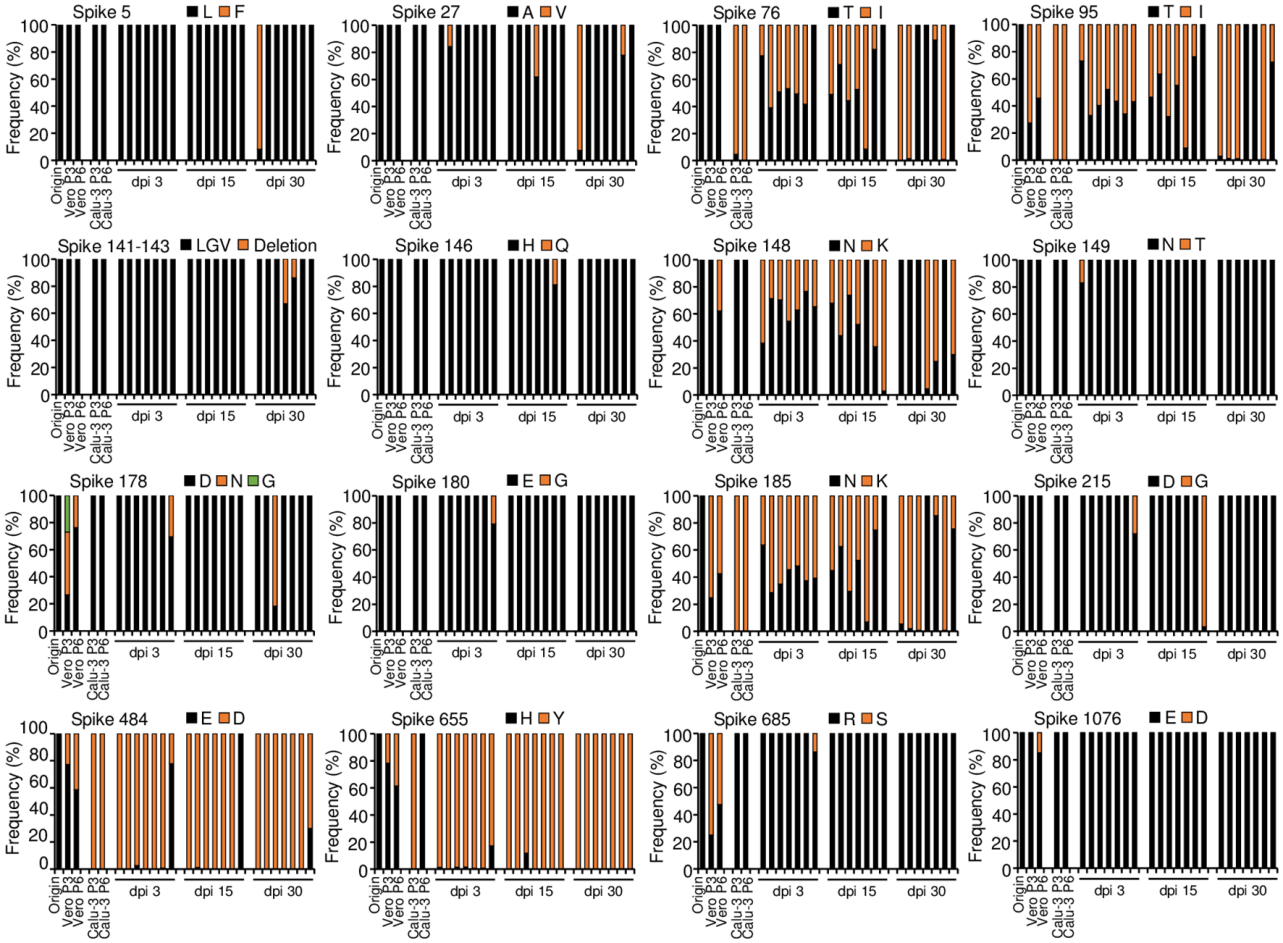
Amino acid mutations of parental SARS-CoV-2 S protein in cells during serial passage and in lung tumor tissues during long-term replication. Calu-3 cells were infected with 0.1 MOI of parental SARS-CoV-2 and then cultured serially 3 or 6 passage at three-day intervals. Calu-3 cells were subcutaneously implanted into the right flank of NRGA mice (n=7 per group). After the tumor volume reached 100 mm^3^, the tumors were inoculated with 1 × 10^6^ pfu of parental SARS-CoV-2. Whole-genome sequences of viruses in cell culture supernatants and supernatants of the tumor homogenates at 3, 15, and 30 dpi were analyzed. Each panel indicates the accumulation and substitutions of mutations at an amino acid position in parental SARS-CoV-2. Origin (parental): amino acid sequences of parental SARS-CoV-2 (**Supplementary Table 1**). Passage 3 (P3) and Passage 6 (P6): amino acid sequences in SARS-CoV-2 after culture in three and six passages, respectively, in Vero E6 cells (Vero) and Calu-3 cells. dpi, days post-infection.

Before the investigation of the long-term SARS-CoV-2 replication in the mouse model, we first confirmed the SARS-CoV-2 mutations in cells during serial passage. We propagated parental SARS-CoV-2 by serial 3 and 6 passage in Vero E6 cells (Vero P3, Vero P6) or Calu-3 cells (Calu-3 P3, Calu-3 P6). We synthesized cDNA using viral RNA isolated from the supernatants of cell culture and conducted an analysis of the sequences by whole-genome deep sequencing. When we compared the results with the sequence of parental virus, there were 6 mutations of S protein in the Vero P3. The same 6 mutations and 2 additional mutations of S protein were found in Vero P6. Most of the mutations showed frequencies of lower than 50% and there were no mutations in other genes (**Figure 2**; **Supplementary Tables 2, 3**). On the other hand, viruses of Calu-3 P3 showed 5 mutations in S protein and 4 among the 5 mutations were maintained in Calu-3 P6. Differently from Vero P3 and Vero P6, most of the S protein mutations showed frequencies of higher than 95%. The mutation H655Y were found in Calu-3 P3 with almost 100% frequency, but completely disappeared in Calu-3 P6 suggesting some disadvantage of the mutation in Calu-3 cells. Viruses of Calu-3 P3 and Calu-3 P6 commonly had one mutation in ORF1ab (**Figure 2**, **Supplementary Tables 2, 3**; **Supplementary Figures 1, 2**). Taken together, most of the mutations were found in S protein with very few additional mutations in other genes when the parental SARS-CoV-2 was propagated in Calu-3 and Vero E6 cells. There were 4 common S protein mutations found in the two different cell culture, but the frequencies of the mutations showed clear discrepancy depending on the cell types.

### 3.3 Appearance of SARS-CoV-2 variants in the mouse xenograft model

To investigate the pattern of mutations in the long-term SARS-CoV-2 replication model, parental SARS-CoV-2 was infected into the xenograft tumors of seven mice and then collected tumor tissues at 3, 15, and 30 dpi. We synthesized viral cDNA using the supernatants of tumor homogenates and analyzed the sequences by whole-genome deep sequencing. The viral sequences showed 15 mutations at 15 residues in the S protein from the xenograft tumors (**Figure 2**). This represents seven more residues compared with the mutations obtained in serial passage cell culture: the mutations L5F, A27V, Del(141-143LGV), H146Q, N149T, E180G, and D215G were found only in the viruses from the tumor tissues, although the frequencies of most of the mutations fluctuated over time in the mice and occurred sporadically among the individual mice. S protein mutations E484D and H655Y were found in all the mice with high frequency (E484D over 97% except one mouse, H655Y over 82%). The mutations T76I, T95I, N148K, and N185K were common and varied in frequency among the mice. The viruses from the tumor tissues had a total of fluctuating 26 mutations in ORF1ab, ORF3a, the M protein, ORF6, ORF7a, and ORF8. However, two mutations were only found in serial passage cell culture (**Supplementary Figures 1, 2**; **Supplementary Table 2, 3**). The S protein mutations found in these mice were previously reported in clinically relevant SARS-CoV-2 variants and SARS-CoV-2 derived from cell culture (**Table 1**). Overall, we confirmed with multiple mice that diverse viral mutations, including some previously reported ones, occurred in the xenograft model, and some mutations were selected *in vivo.* Therefore, it is likely that the model can provide evolution of variants through *in vivo* long-term replication.

**Table 1:** Summary of the S protein mutations found in this study.

Classification*	Position	Mutation	Occurrence in this study**		Occurring in clinical isolates	Occurring in clinical isolates at homologous position		References
			in cell culture	in mice				
Major mutations	27	A27V	–	+		A27S	Omicrons	Sonnleitner et al., 2022
	76	T76I	–	+	Lambda			Sonnleitner et al., 2022
	95	T95I	+	+	Omicron, Mu			Guruprasad, 2021; Puray-Chavez et al., 2021
	148	N148K	–	+				Guruprasad, 2021
	185	N185K	–	+	Various regions (2019-2020)			Choi et al., 2020
	484	E484D	+	+		E484K	Beta, Gamma, Mu, Eta	Bashor et al., 2021; Guruprasad, 2021; Puray-Chavez et al., 2021
						E484Q	Kappa	
						E484A	Omicrons	
	655	H655Y	+	+	Gamma, Omicrons			Guruprasad, 2021; Puray-Chavez et al., 2021
Minor mutations	5	L5F	–	+	Iota			Guruprasad, 2021
	141-143	Del (141-143 LGV)	–	+		G142D	Delta, Omicrons	Puray-Chavez et al., 2021
						Del(G142)	Omicrons, Eta	Puray-Chavez et al., 2021
						Del(Y144)	Omicron BA.1, Eta	Puray-Chavez et al., 2021
						Del(141-144)	Immunocompromised patient, 20B	Ou et al., 2022
	146	H146Q	–	+	Omicron 22F(XBB), 23A(XBB.1.5)	H146K H146del	Omicron 22F(XBB), 23A(XBB.1.5)	Puray-Chavez et al., 2021
	149	N149T	–	+	Omicron 23A(XBB.1.5)			Muñoz-Fontela et al., 2022
	178	D178G	+	–	Gamma plus, P.1.12 (rare)			Bashor et al., 2021
	178	D178N	+	+	North America (2019-2020)			Harrison et al., 2020
	180	E180G	–	+		E180G		Pires De Souza et al., 2022
	215	D215G	–	+	Beta			Guruprasad, 2021
	685	R685S	+	+				Harrison et al., 2020

*When the mutations were found in three or more mice with over 10% frequency, the mutations were classified to major mutations.

**When the mutations were found in cell culture or in mice, it was indicated as +.

### 3.4 Preparation and characterization of viral isolates from tumors infected with parental SARS-CoV-2

**Figure 3:**
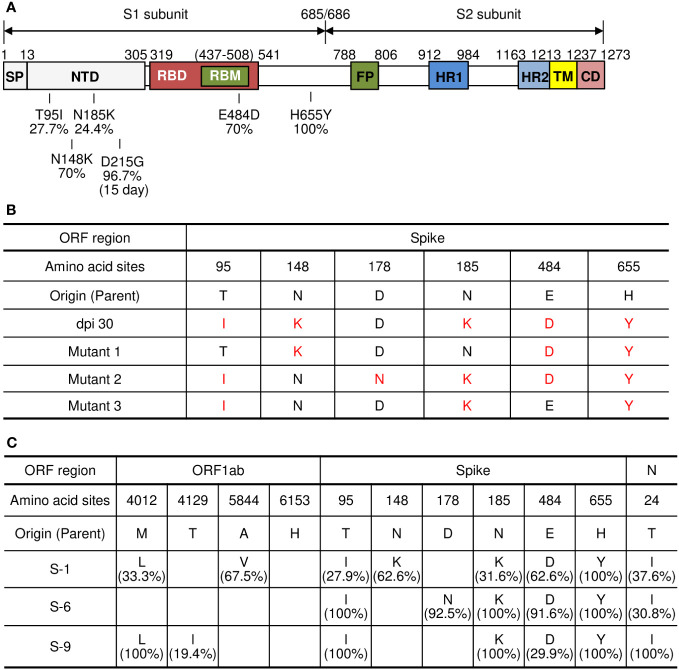
Viral clone isolation from plaques and analysis of amino acid mutations. **(A)** Schematic diagram of amino acid mutations in the S protein of parental SARS-CoV-2. The mutations were analyzed by whole-genome sequencing of SARS-CoV-2 virus isolated from the tumor #7 at 30 dpi as shown in **Supplementary Table 3**. **(B)** Analysis of amino acid mutations in the S protein of parental SARS-CoV-2 and its variants in lung tumor. cDNA was synthesized from the pool of viruses in the supernatants of SARS-CoV-2 parental virus-infected tumor homogenates obtained at 30 dpi. The sequences of the S1 subunit region of the S protein were cloned and analyzed by direct DNA sequencing. **(C)** Viral clone isolation from plaques and analysis of amino acid mutations. Viral isolates were isolated from plaques derived from supernatants of the tumor infected with parental SARS-CoV-2 at 30 dpi. Fifteen viral isolates collected and then cDNA was synthesized from each clone. The S1 subunit region of the S protein was further cloned and analyzed by direct DNA sequencing. Three selected viral isolates (S-1, S-6, S-9) were collected, and their whole-genome sequences were analyzed. Each panel indicates the accumulation and substitutions of mutations at an amino acid position in parental SARS-CoV-2. Origin (Parent): amino acid sequences of parental SARS-CoV-2 (**Supplementary Table 1**). dpi, days post-infection.

**Figure 4:**
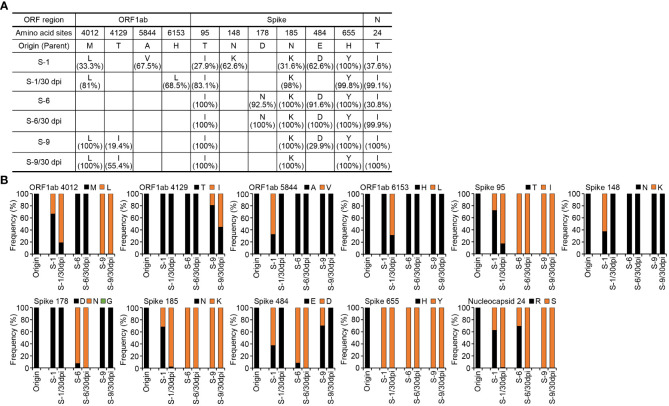
Analysis of amino acid mutations in the viruses obtained by re-infection in lung tumor xenograft mouse model. The tumors derived from Calu-3 cells were infected with 1 x 10^6^ pfu of the viral isolates (S-1, S-6, S-9) from **Figure 3**. Tumor tissues were excised at 30 dpi and supernatants of the tumor homogenates were collected. Viral RNAs were extracted from the supernatants and the whole-genome sequences were analyzed. **(A)** Analysis of amino acid mutations in the three viral isolates (S-1, S-6, S-9) and viruses from tissue homogenates obtained at 30 dpi after re-infection (S-1/30 dpi, S-6/30 dpi, S-9/30 dpi). **(B)** Each panel indicates the accumulation and substitutions of mutations at an amino acid position in the viral isolates and their derivative viruses. Origin (Parent): amino acid sequences of parental SARS-CoV-2 (**Supplementary Table 1**). dpi, days post-infection.

Next, we aimed to analyze the S protein sequences of individual viruses obtained from the mouse xenografts at 30 dpi in **Figures 2**, **3A**. We selected the xenograft of mouse #7 as a specific target to analyze. As most of the confirmed mutations were located on the S1 subunit of the S protein, we first cloned the cDNA sequences of that region and analyzed them by further direct DNA sequencing to overview the mutation composition in specific viruses obtained from the xenograft tumor. As shown in **Figure 3B**, the sequencing of the S1 subunit revealed three different mutants (mutants 1, 2, and 3) based on the combinations of mutations across six amino acid residues. To specifically characterize SARS-CoV-2 variants that emerged in the xenograft tumor, we randomly selected 15 viral isolates from individual plaques after plaque formation assays. Then, we identified three isolates (S-1, S-6, and S-9) corresponding to the three mutants based on the S1 subunit sequences and performed whole-genome deep sequencing on the isolates (**Figure 3C**). The S1 subunit sequences of the three isolates (S-1, S-6, and S-9) showed that the virus isolates are composed of various sequence combinations. None the less, the results showed that the three isolates overall had the expected S mutations along with three mutations in the ORF1ab and a mutation in the N protein (**Figure 3C**). All three isolates had the H655Y mutation at or near 100% frequency, no presence of the R685S and T1076A mutation, and the N protein mutation T24I at a frequency of at least 30%. To further investigate emerging mutations from the three isolates, we re-infected each viral isolate to the mouse xenograft tumors and analyzed the virus sequences at 30 dpi. Although there were minor mutations in in the viruses derived from S-1 and S-6, the viral sequences were mostly conserved with some change in the frequency (**Figure 4**). Therefore, the viral sequences of the three isolates seem largely stabilized in the specific environment of our mouse xenograft model.

**Figure 5:**
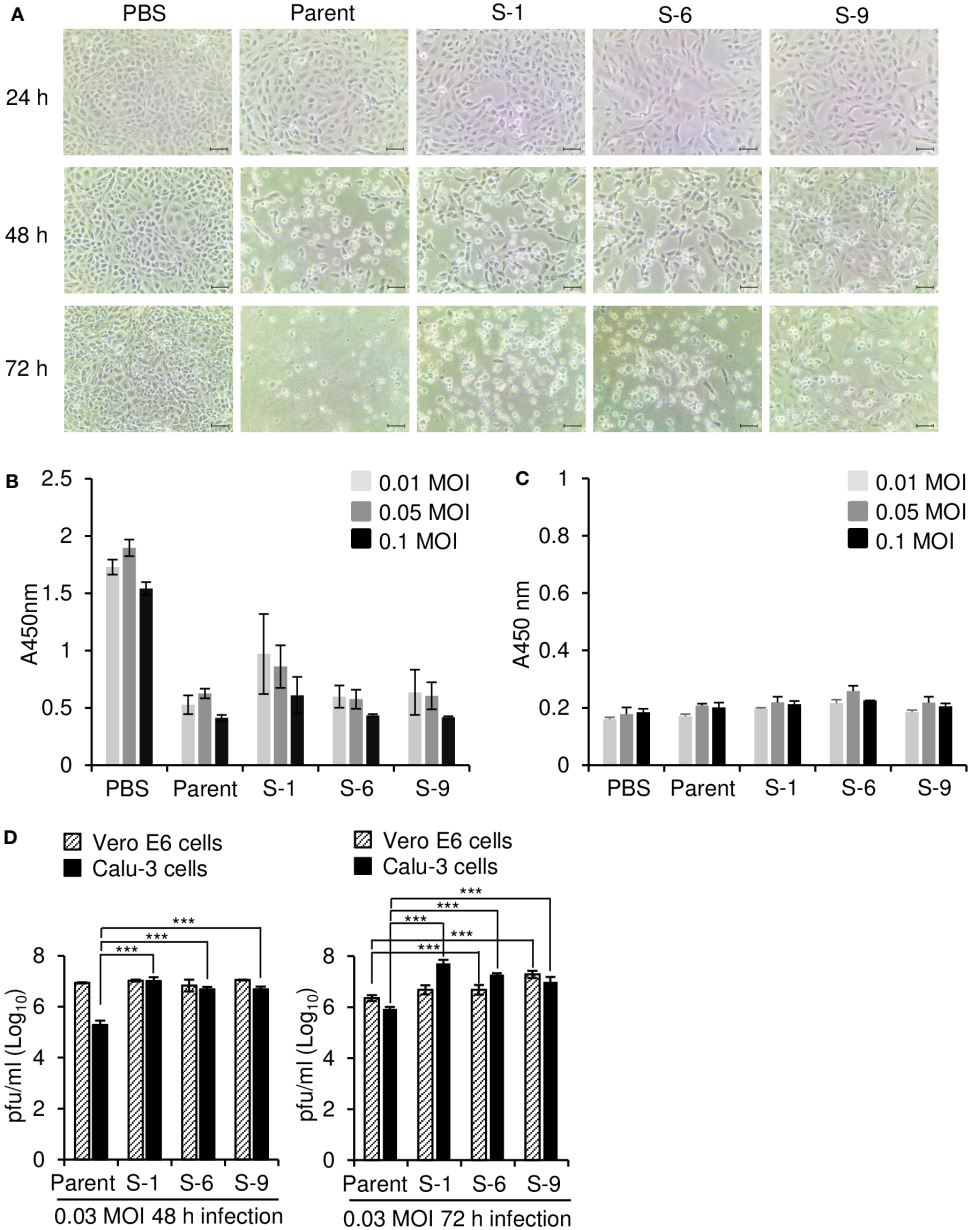
Differential replication of parental SARS-CoV-2 and its variants in Vero E6 and Calu-3 cells. **(A)** Microscopic observation of SARS-CoV-2–infected Vero E6 cells. Vero E6 cells were infected with parental SARS-CoV-2 or its variants at 0.05 multiplicity of infection (MOI). At the indicated times after infection, the cells were observed with a bright light microscope. Scale bar, 50 μm. **(B, C)** Cell viability assay. Vero E6 **(B)** and Calu-3 **(C)** cells were infected with parental SARS-CoV-2 or its variants (n = 3) at the indicated MOI. After 72 h, cell viability was determined by CCK-8 assay. **(D)** Replication of SARS-CoV-2 in Vero E6 and Calu-3 cells. Vero E6 and Calu-3 cells (2 × 10^5^ cells/well on 24-well plates) were infected with parental SARS-CoV-2 or its variants (S-1, S-6, S-9) in PBS at an MOI of 0.03 for 1 h (n = 3). At the indicated times after infection, supernatants were collected and the virus titers were determined by plaque assay. The *P* values were determined by two-sided unpaired *t*-test. ***p < 0.001. Representative data are shown from at least 2 independent experiments **(A–D)**.

To further compare the isolates derived from the xenograft tumor with the parental virus, we investigated cytopathic effects of the isolates in Vero E6 cells and Calu-3 cells. In Vero E6 cells, the parental virus and isolates S-1, S-6, and S-9 induced clear cytopathic effects to a similar extent (**Figures 5A, B**). However, no cytopathic effects were induced by any of the viruses in Calu-3 cells (**Figure 5C**), as we previously reported (Park et al., 2021a). When we measured virus replication 48 h after infection by plaque assay, there was no difference between the parental SARS-CoV-2 and the three isolates (S-1, S-6, S-9) in Vero E6 cells; however, all three isolates showed higher virus titers than the parental SARS-CoV-2 in Calu-3 cells (**Figure 5D**). At 72 h after infection, we found higher replication of S-6 and S-9 compared to the parental SARS-CoV-2 in Vero E6 cells. Taken together, the viral titers seem to reach plateau at 48 h after infection and the titers of parental virus more quickly decrease in Vero E6 cells. Considering that the parental virus replicates less in Calu-3 cells than in Vero E6 cells, the effects of mutation could be monitored more easily in Calu-3 cells. It is likely that the mutations contributed to the increased replication in Calu-3 cells.

### 3.5 Properties of SARS-CoV-2 variants in the K18-hACE2 mouse model

**Figure 6:**
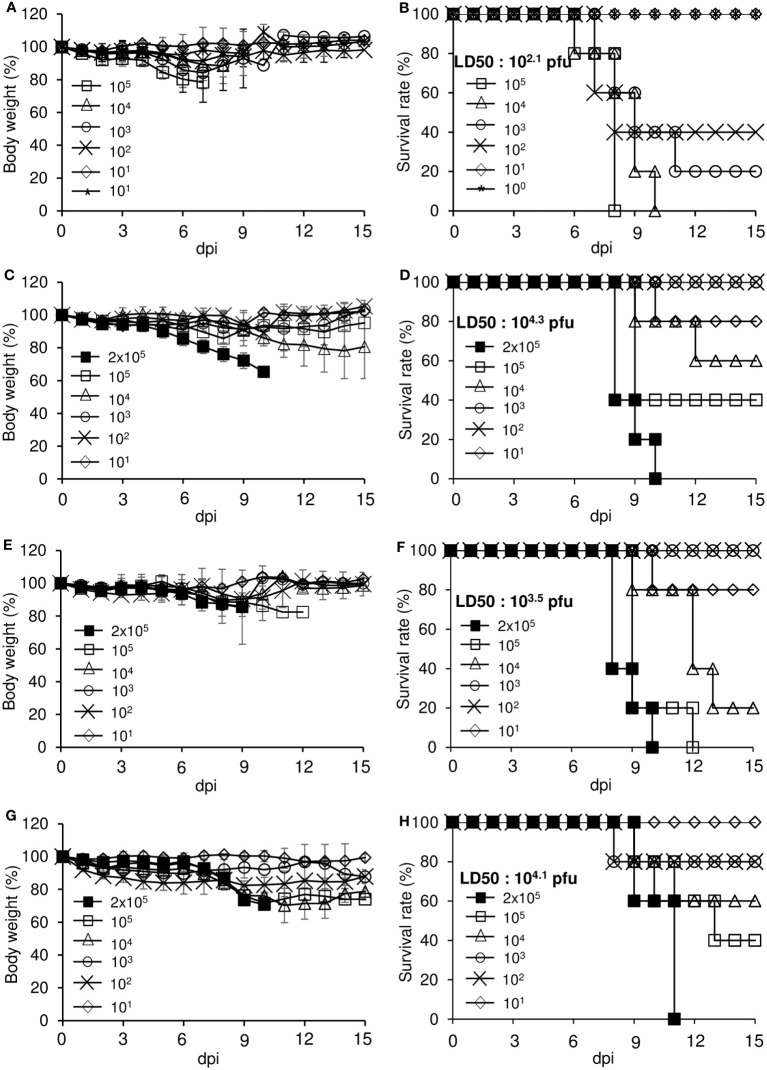
Determination of LD_50_ for each virus in K18-hACE2 mice. Eight-week-old male hemizygous K18-hACE2 mice (n = 5 per group) were intranasally inoculated with the indicated titers of parental SARS-CoV-2 or its variants. The mice were monitored daily for clinical signs and measured for body weight **(A, C, E, G)** and survival **(B, D, F, H)** for up to 15 days. **(A, B)** Parental SARS-CoV-2. **(C, D)** S-1 isolate. **(E, F)** S-6 isolate. **(G, H)** S-9 isolate. dpi, days post-infection.

**Figure 7:**
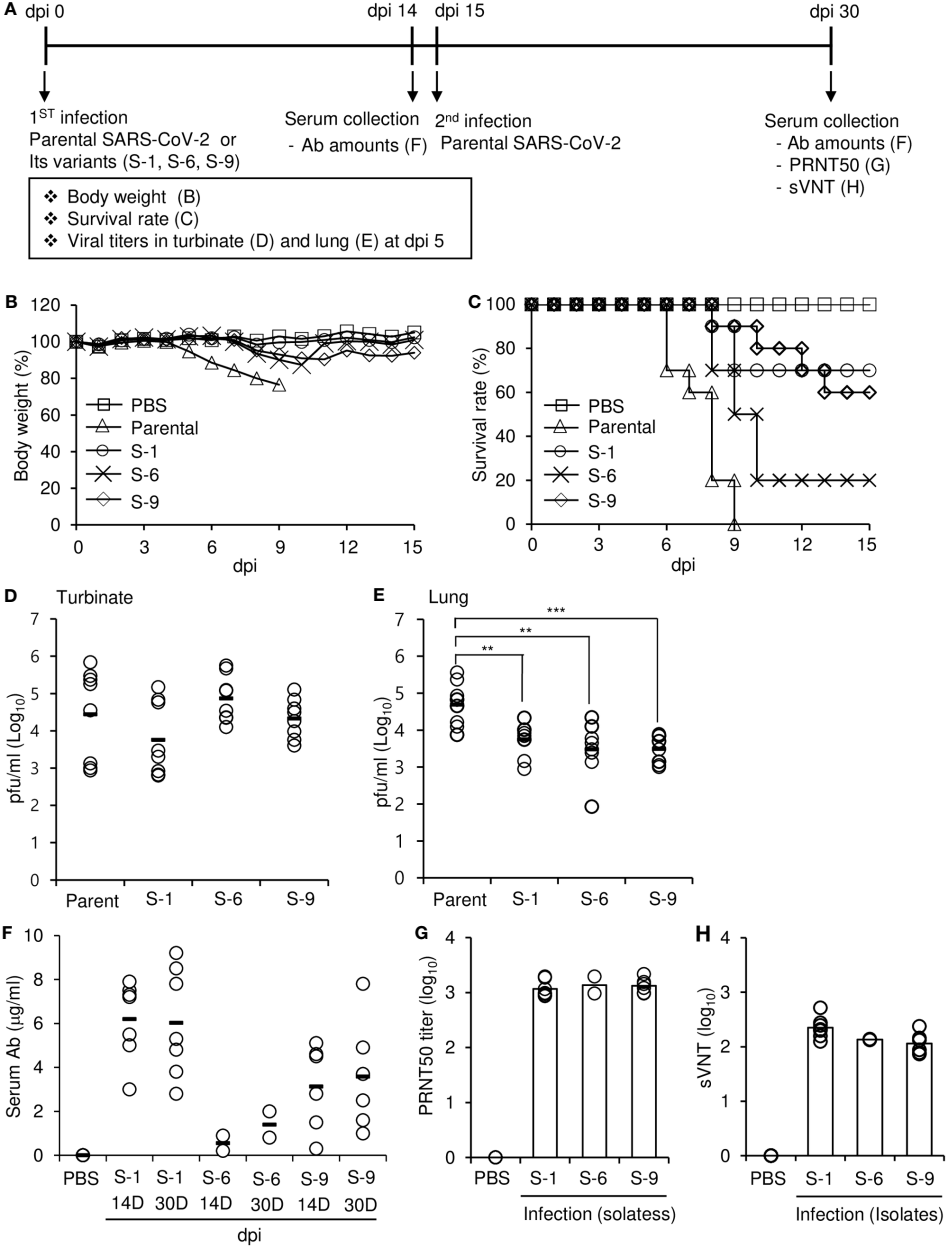
Effects of parental SARS-CoV-2 and its variants on hACE2 transgenic mice (B6.Cg-Tg(K18-ACE2)2Prlmn/J). **(A)** Schematic diagram of the experiments. **(B–E)** Hemizygous K18-hACE2 mice were intranasally infected with 3 × 10^4^ pfu/mouse of parental SARS-CoV-2 or its variants. Body weight **(B)** and survival **(C)** were measured for 15 days after intranasal infection (n = 10 per group). dpi, days post-infection. Turbinates **(D)** and lungs **(E)** were collected 5 days after intranasal infection (n = 8 per group). Viral loads in turbinate and lung homogenates were measured by plaque assay. The *P* values were determined by two-sided unpaired *t*-test. **p < 0.05, ***p < 0.001. **(F–H)** Hemizygous K18-hACE2 mice were intranasally infected with 3 × 10^4^ pfu/mouse of parental SARS-CoV-2 or its variants (n = 10 per group). Serum was collected from surviving mice 14 days (14D) after infection. Fifteen days after the first infection, the surviving mice were intranasally challenged with 3 × 10^4^ pfu/mouse of parental SARS-CoV-2. Serum was collected 15 days (30D) after the challenge. **(F)** The amounts of parental SARS-CoV-2 S protein RBD-specific IgG in the serum were determined by ELISA. **(G)** The PRNT_50_ of the serum collected at 15 days (30D) after the challenge was determined by plaque assay. **(H)** The sVNT of the serum collected at 15 days (30D) after the challenge was determined against parental SARS-CoV-2.

To estimate the lethality of variants that arose during long-term replication in the mouse xenograft, we infected K18-hACE2 mice with various concentrations of the three isolates derived from the xenograft tumor and measured body weight and survival rate to determine the median lethal dose (LD_50_) (**Figure 6**). The body weights of mice infected with the viruses gradually decreased during the experimental period, especially at higher concentrations of the virus. The mice were more sensitive to the parental SARS-CoV-2 than the isolates S-1, S-6, and S-9. The LD_50_ of isolates S-1, S-6, and S-9 were 2.0 × 10^4^, 3.2 × 10^3^, and 1.3 × 10^4^, respectively, whereas the LD_50_ of the parental SARS-CoV-2 was 1.3 × 10^2^. To further estimate the virulence properties of the isolates, we infected K18-hACE2 mice with parental virus or one of the three isolates and monitored them according to the experimental scheme shown in **Figure 7A**. When the mice were infected with a lethal dose of parental SARS-CoV-2 (3 × 10^4^ pfu), loss of body weight was clearly detected, and all the mice died by 9 days after infection (**Figures 7B, C**). By contrast, when the mice were infected with the same dose of one of the isolates, the resulting weight loss was relatively mild, and some of the mice survived. When we measured virus titers 5 days after infection, the virus titers in turbinate were similar for the parental SARS-CoV-2 and the isolates (S-1, S-6, S-9) (**Figure 7D**), but the titers in the lungs were lower for the isolates than for the parental SARS-CoV-2 (**Figure 7E**). At 14 dpi, the sera of surviving mice infected with each of the isolates contained IgG reactive to the parental SARS-CoV-2 S protein RBD (**Figure 7F**), suggesting that infection with the isolates induced production of antibodies that were cross-reactive to the parental virus.

To investigate whether prior infection with one of the isolates could protect the mice against subsequent challenges with the parental virus, we challenged the surviving mice with a potentially lethal dose of parental virus and found that all the mice were still alive 15 days after the challenge. At 15 days after the challenge with parental virus, the amounts of serum antibodies were similar to or slightly higher than those before the challenge, suggesting that there was no boosting effect. However, PRNT_50_ and sVNT assays showed that the mice had substantial amounts of neutralizing antibodies against the parental virus in their serum (**Figures 7G, H**). Interestingly, the ratio of neutralizing antibodies to total anti-RBD IgG were different among isolates (**Figure 7F** vs **Figures 7G, H**): S-6 isolate had a higher value. It is likely that the sequence of S-6 somehow contributes to the production of antibodies with higher neutralizing activity against parental SARS-CoV-2. These results suggest that the lethality of the isolates was lower than that of the parental SARS-CoV-2, and infection with the isolates provided protective immunity against subsequent challenge with the more lethal parental virus.

## 4 Discussion

RNA viruses have high mutation rates because of the low fidelity of RNA-dependent RNA polymerase. These viruses also encounter host genetic variation and diverse cellular microenvironments during infection. During the last 3 years, several SARS-CoV-2 variants have appeared and transmit around the globe. Several hypotheses were suggested to explain this emergence of SARS-CoV-2 variants, including persistent infection in immunocompromised patients (Avanzato et al., 2020; Choi et al., 2020; Borges et al., 2021), simultaneous infection and recombination of variants in unvaccinated or immunocompromised individuals (Lueking et al., 2022; Markov et al., 2023) and transmission between humans and animals resulting in accelerated evolution (Bashor et al., 2021). We are interested in the first hypothesis: persistent infection of SARS-CoV-2.

There are animal models to recapitulate symptoms and immune responses after SARS-CoV-2 infection, including human ACE2-transgenic mice, hamsters, ferrets, and non-human primates (Muñoz-Fontela et al., 2022). These models are useful to study the pathogenesis, virulence, and immune escape of variants of concern and the efficacy of vaccines and therapeutics against these variants. For persistent infection, there are two factors required: long-term replication and reduced immunity. Recently, a humanized mouse model for persistent infection of SARS-CoV-2 was established by delivering human ACE2 to mouse lung tissues using adeno-associated virus and it was proved to recapitulate innate and adaptive human immune responses against SARS-CoV-2 infection up to 28 days after infection (Sefik et al., 2022). In this study, we focused on the effect of long-term replication of SARS-CoV-2 using xenograft tumors established with Calu-3 cells in severely immunodeficient mice and investigated variants emerged in the model. Our xenograft infection model is limited in its ability to simulate persistent infections due to the significant lack of immunity in NRGA mice. However, it serves as a beneficial *in vivo* incubator model that is easily accessible and allows for the emergence of SARS-CoV-2 variants following extended replication periods.

The first evidence suggesting that our model enables long-term replication of SARS-CoV-2 *in vivo* is the continuous production of viruses in the tumor tissues. When we established xenografts with Calu-3 cells, the engrafted mice survived without prominent pathophysiological phenotype during the experimental period. Furthermore, when the xenografts were infected with SARS-CoV-2, the mice showed no prominent side effects of virus infection, although high titers of virus were present in the tumor tissues for 30 days, which was the endpoint of our experiment. The mice with the xenograft tumors even without infection (mock infection) gradually face with necrosis and release of exudates in the tumors after 30 days, therefore we did not measure virus titers after 30 dpi. However, if we find better tumor model, long-term replication in the mouse xenografts may continue much longer, as it does in human patients with SARS-CoV-2 infection (Avanzato et al., 2020; Borges et al., 2021; Clark et al., 2021; Lueking et al., 2022). We believe the selection of appropriate cells that do not suffer cytopathic effects due to SARS-CoV-2 infection was the most important factor in our model. As we reported previously, Calu-3 cells replicated continuously without apoptosis for 3 days after SARS-CoV-2 infection, in contrast to Vero cells that displayed cytopathic effects after infection (Park et al., 2021a). Calu-3 cells are cancer cells derived from the human lung, a primary target organ of SARS-CoV-2, but SARS-CoV-2 has also been found in other organs in humans including the gastrointestinal tract, liver, and brain (Pires De Souza et al., 2022). Furthermore, extensive autopsy data suggest that SARS-CoV-2 causes systemic infection in humans with a broad range of severity and can persist in various organs for several months (Stein et al., 2022). As human cell lines such as Caco-2 colorectal adenocarcinoma cells, Huh-7 liver cancer cells, and U251 glioblastoma cells are susceptible to SARS-CoV-2 without cytopathic effects (Pires De Souza et al., 2022), we speculate that other cell lines can be used in xenograft infection models to provide more insights into persistent replication in different human tissues.

The gene for the S protein covers only about 12.8% of the SARS-CoV-2 genome; however, 60% of the mutations that differentiate the Omicron variant from the parental strain are in the S protein (Jung et al., 2022). This trend was also found in our results. Several mutations in the viral genomes isolated from multiple mice at 30 dpi were mostly found in the S protein, especially the S1 region, and most of these mutations were previously reported in natural variants or variants obtained by cell culture. Some major mutations reached high frequency in the xenograft infections, whereas others were displaced by the original sequences. For example, all viral isolates isolated from the xenografts showed the S protein mutation H655Y and no mutation at residue S685. Therefore, we conclude that there were continuous changes in the virus pool in the mouse xenografts with some unique changes. In this context, we propose a possibility that our *in vivo* long-term replication model recapitulates natural infection for long period, at least in part; however, the important key mutation D614G was not found in our study, which was the same as in a previous investigation using cell culture (Zhang et al., 2020). The D614G mutation results in higher infectious titers by increasing the stability of the S protein and promoting higher incorporation of S protein into virions without changing the affinity of the S protein for hACE2 (Zhang et al., 2020; Plante et al., 2021). Therefore, we speculate that the probability of this mutation is very low, but it was somehow selected in humans but not in mice. It is also possible that our model has a limitation as the xenografts are in a severely immunodeficient mouse host. This situation can’t provide any selective pressure with which viruses usually faces in the general host. In the near future, we are planning to inject antiviral drugs or therapeutic antibodies to our model and investigate their effects on the emergence of variants.

Mutations in the S protein are important in the infection, pathogenicity, and immune escape of SARS-CoV-2 variants (Zhang et al., 2020; Plante et al., 2021; Carabelli et al., 2023). The functional effects of the identified mutations on virus properties are largely unknown, except for E484D, R685S, and H655Y. The E484D mutation was implicated in ACE2-independent entry of SARS-CoV-2 into cells.^17^ Mutations at this site (E484A, E484D, E484G, and E484K) contribute to escape from neutralization by antibodies from convalescent plasma (Harvey et al., 2021). The SARS-CoV-2 pseudovirus containing E484D/R685S double mutant was ACE2-dependent suggesting the suppressive effect of R685S mutation against E484D (Puray-Chavez et al., 2021). The H655Y mutation is expected to enhance the endosomal entry pathway and contribute to the low pathogenicity of the Omicron variant (Hu et al., 2022). Considering that all viral isolates obtained by the long-term replication in mouse xenografts had the mutation H655Y with no R685S mutation, it is likely that our model majorly induces ACE2-dependent human adaptation of SARS-CoV-2 resulting in variants with lowered pathogenicity. Although we focused on S protein mutations, we also found several mutations in the N protein, ORF1ab, ORF3a, ORF6, ORF7a, and ORF8. Considering that mutations modulating protein levels of innate immunity antagonists such as the N protein, ORF9b, and ORF6 enhance viral escape from host innate immunity (Thorne et al., 2022), our results might provide clues about additional genes and mechanisms contributing to the properties of variants. In addition, further investigation of the variants with multiple mutations will provide insights into the combined effects of the mutations. Such efforts may help to predict the properties of emerging variants once a genomic sequence is obtained without any further knowledge of the variants. In fact, when we analyzed individual viral isolates, they had different combinations of mutations and showed higher replication in Calu-3 cells and decreased lethality in mouse challenge experiments compared with the parental SARS-CoV-2. These results support general speculation that SARS-CoV-2 variants acquire mutations that promote increased replication and decreased lethality as they adapt to their human host. Taken together, our results suggest that Calu-3 xenograft mice can be used as a model to study the effect of long-term replication of parental SARS-CoV-2 *in vivo* and investigate the properties of its variants. There is a possibility that the trend of emerging mutations can be different depending on the virus strain. We will apply the same strategy with other variants such as Delta and Omicrons in the future and the results may provide better understanding of the emergence of variants.

## Data availability statement

The datasets presented in this study can be found in online repositories. The names of the repository/repositories and accession number(s) can be found in the article/**Supplementary Material**.

## Ethics statement

The animal study was approved by Hallym University Institutional Animal Care and Use Committee. The study was conducted in accordance with the local legislation and institutional requirements.

## Author contributions

DK: Conceptualization, Data curation, Formal Analysis, Investigation, Methodology, Validation, Writing – original draft. JK: Conceptualization, Data curation, Formal Analysis, Validation, Writing – original draft, Methodology. MK: Data curation, Formal Analysis, Methodology, Validation, Writing – original draft. HP: Data curation, Formal Analysis, Methodology, Validation, Writing – original draft. SP: Data curation, Formal Analysis, Methodology, Validation, Writing – original draft. SM: Formal Analysis, Validation, Writing – original draft. KB: Data curation, Formal Analysis, Methodology, Writing – original draft. BMK: Formal Analysis, Methodology, Writing – original draft. SK: Formal Analysis, Methodology, Writing – original draft. M-SP: Conceptualization, Data curation, Formal Analysis, Funding acquisition, Investigation, Project administration, Supervision, Validation, Writing – original draft. YL: Conceptualization, Data curation, Formal Analysis, Funding acquisition, Investigation, Project administration, Supervision, Validation, Writing – original draft, Writing – review & editing. H-JK: Conceptualization, Data curation, Formal Analysis, Funding acquisition, Investigation, Project administration, Supervision, Validation, Writing – original draft, Writing – review & editing.
