# Supplementary material for: Analysis of spike protein variants evolved in a novel in vivo long-term replication model for SARS-CoV-2
Source: Front Cell Infect Microbiol. 2023 Nov 3;13:1280686. doi: 10.3389/fcimb.2023.1280686 (PMC10655031; doi:10.3389/fcimb.2023.1280686)
Supplement: Supplementary file 1 [file DataSheet_1.pdf]

Supplementary Material

Supplementary Figure 1
Supplementary Figure 2
Supplementary Table 1
Supplementary Table 2
Supplementary Table 3
Data Availability Statement

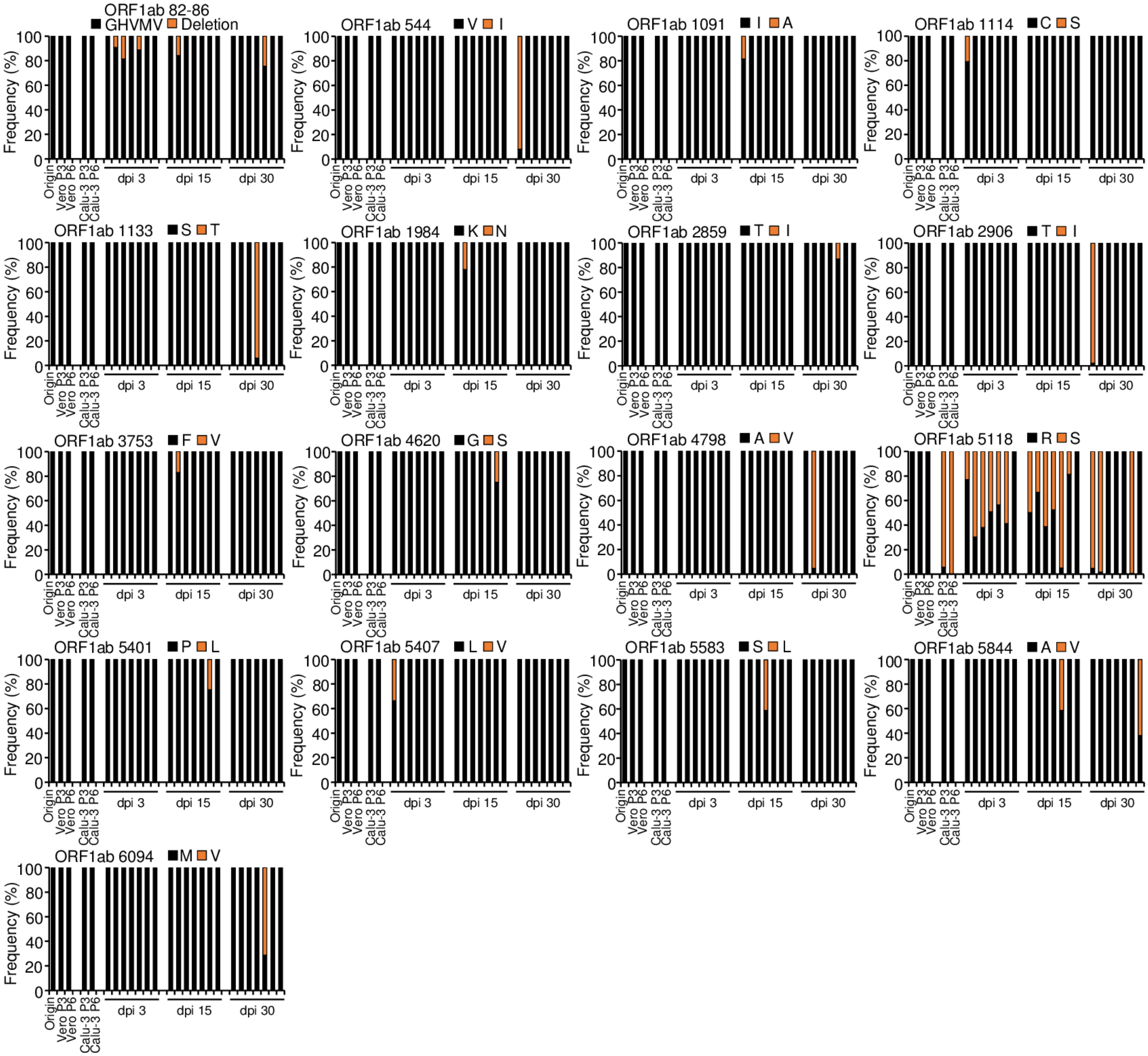

Supplementary Figure 1. Frequencies and substitutions of amino acid sequences in parental SARS-CoV-2 ORF1ab in cells during serial passage and in lung tumor tissues during long-term replication. Calu-3 cells were infected with 0.1 MOI of parental SARS-CoV-2 and then cultured serially 3 or 6 passage at three-day intervals. Calu-3 cells were subcutaneously implanted into the right flank of NRGA mice (n=7 per group). After the tumor volume reached 100 mm^3, the tumors were inoculated with 1×10^6 pfu of parental SARS-CoV-2. Whole-genome sequences of viruses in cell culture supernatants and supernatants of the tumor homogenates at 3, 15, and 30 dpi were analyzed. Each panel indicates the accumulation and substitutions of mutations at an amino acid position in parental SARS-CoV-2. Origin (parental): amino acid sequences of parental SARS-CoV-2 (Supplementary Table 1). Passage 3 (P3) and Passage 6 (P6): amino acid sequences in SARS-CoV-2 after culture in three and six passages, respectively, in Vero E6 cells (Vero) and Calu-3 cells. dpi, days post-infection.

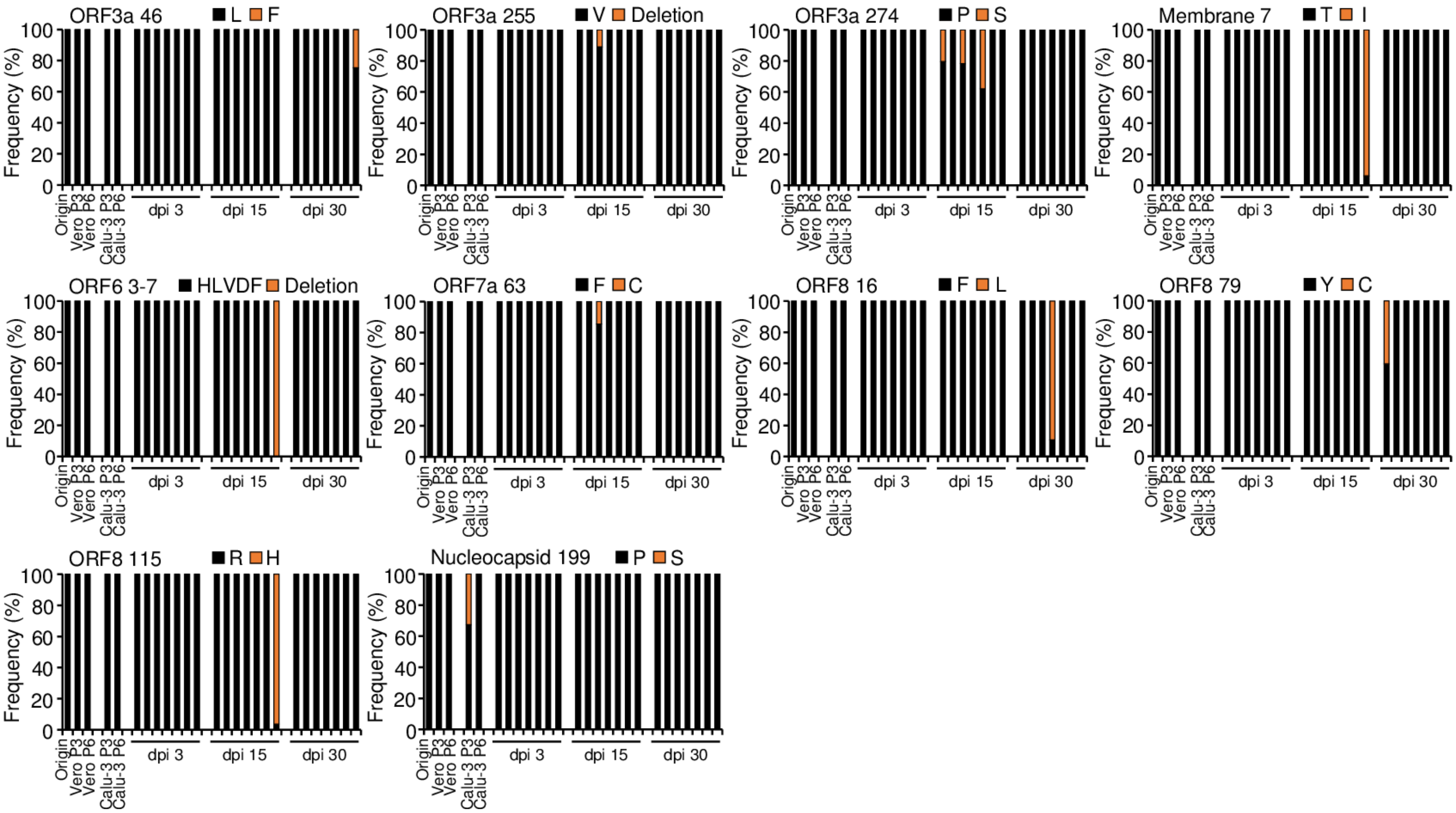

Supplementary Figure 2. Frequencies and substitutions of amino acid sequences in parental SARS-CoV-2 ORF3a, ORF7a, and ORF8 in cells during serial passage and in lung tumor tissues during long-term replication. Calu-3 cells were infected with 0.1 MOI of parental SARS-CoV-2 and then cultured serially 3 or 6 passage at three-day intervals. Calu-3 cells were subcutaneously implanted into the right flank of NRGA mice (n=7 per group). After the tumor volume reached 100 mm^3, the tumors were inoculated with 1×10^6 pfu of parental SARS-CoV-2. Whole-genome sequences of viruses in cell culture supernatants and supernatants of the tumor homogenates at 3, 15, and 30 dpi were analyzed. Each panel indicates the accumulation and substitutions of mutations at an amino acid position in parental SARS-CoV-2. Origin (parental): amino acid sequences of parental SARS-CoV-2 (Supplementary Table 1). Passage 3 (P3) and Passage 6 (P6): amino acid sequences in SARS-CoV-2 after culture in three and six passages, respectively, in Vero E6 cells (Vero) and Calu-3 cells. dpi, days post-infection.

Supplementary Table 1. List of accession numbers for SARS-CoV-2 proteins based on GenBank: MW466791.1.
(https://www.ncbi.nlm.nih.gov/protein?LinkName=nuccore_protein&from_uid=1955390757)

Proteins	Amino acid no.	Accession no.
ORF1ab	7096	QQO75521.1
ORF1a	4405	QQO75522.1
Spike	1273	QQO75523.1
ORF3a	275	QQO75524.1
Envelope	75	QQO75525.1
Membrane	222	QQO75526.1
ORF6	61	QQO75527.1
ORF7a	121	QQO75528.1
ORF7b	43	QQO75529.1
ORF8	121	QQO75530.1
Nucleocapsid	419	QQO75531.1
ORF10	38	QQO75532.1

Supplementary Table 2. Frequency of mutations in parental SARS-CoV-2 ORF1ab in cells during serial passage and in lung tumor tissues during long-term replication.

ORF region		ORF1ab																
Amino acid sites		82-86	544	1091	1114	1133	1984	2859	2906	3753	4620	4798	5118	5401	5407	5583	5844	6094
Origin		GHVMV	V	I	C	S	K	T	T	F	G	A	R	P	L	S	A	M
Vero P3																		
Vero P6																		
Calu-3 P3													S (94.2%)					
Calu-3 P6													S (100%)					
dpi 3	#1				S (20.6%)								S (23%)		V (33.7%)			
	#2	Deletion (9.3%)											S (69.6%)					
	#3	Deletion (18.6%)											S (61.8%)					
	#4												S (49.1%)					
	#5	Deletion (11.3%)											S (43.5%)					
	#6												S (58.7%)					
	#7																	
dpi 15	#1			A (18.5%)									S (49.8%)					
	#2	Deletion (15.7%)					N (22%)			V (17%)			S (33.2%)					
	#3												S (61.2%)					
	#4												S (47.6%)			L (41.2%)		
	#5												S (94.9%)				V (41.5%)	
	#6												S (18.7%)	L (24.7%)				
	#7										S (25%)							
dpi 30	#1								I (97.8%)				S (95.2%)					
	#2											V (95.4%)	S (98.2%)					
	#3																	
	#4		I (91.9%)			T (94%)												
	#5	Deletion (24.4%)						I (13%)										V (71.2%)
	#6												S (99.3%)					
	#7																V (61.8%)	

Supplementary Table 3. Frequency of mutations in parental SARS-CoV-2 in cells during serial passage and in lung tumor tissues during long-term replication (Spike, ORF3a, ORF7a, ORF8).

ORF region		Spike																ORF3a			M	ORF6	ORF7a	ORF8			N
Amino acid sites		5	27	76	95	141-143	146	148	149	178	180	185	215	484	655	685	1076	46	255	274	7	3-7	63	16	79	115	199
Origin		L	A	T	T	LGV	H	N	N	D	E	N	D	E	H	R	T	L	V	P	T	HLVDF	F	F	Y	R	P
Vero P3					I (72.5%)					N (46.3%) G (27%)		K (75.3%)		D (22.8%)	Y (21.7%)	S (75%)											
Vero P6					I (54.4%)			K (37.8%)		N (23.7%)		K (57.3%)		D (41.4%)	Y (38.6%)	S (52.4%)	A (14.7%)										
Calu-3 P3				I (95.3%)	I (99.9%)							K (99.9%)		D (99.9%)	Y (99.6%)												S (32.7%)
Calu-3 P6				I (99.9%)	I (99.9%)							K (100%)		D (99.8%)													
dpi 3	#1			I (22.5%)	I (26.9%)			K (61.6%)	T (17%)			K (36.2%)		D (100%)	Y (98.7%)												
	#2		V (15.7%)	I (60.9%)	I (67%)			K (28.8%)				K (71.3%)		D (100%)	Y (100%)												
	#3			I (49.1%)	I (59.6%)			K (29.7%)				K (65.2%)		D (97.4%)	Y (98.5%)												
	#4			I (46.7%)	I (47.9%)			K (45.3%)				K (54.5%)		D (100%)	Y (98.4%)												
	#5			I (50.7%)	I (56.5%)			K (37.1%)				K (51.8%)		D (100%)	Y (99.3%)												
	#6			I (58.3%)	I (65.8%)			K (23.4%)				K (62.7%)		D (99.2%)	Y (99.1%)												
	#7				I (56.8%)			K (34.7%)		N (30.4%)	G (20.8%)	K (60.8%)	G (27.9%)	D (22.1%)	Y (82.8%)	S (13.6%)											
dpi 15	#1			I (51.1%)	I (53.4%)			K (32.2%)				K (55%)		D (100%)	Y (100%)					S (20.3%)							
	#2			I (29%)	I (36.5%)			K (56.1%)				K (37.3%)		D (98.9%)	Y (100%)												
	#3			I (55.6%)	I (67.9%)			K (26.3%)				K (70.6%)		D (100%)	Y (88.1%)				Deletion (11%)	S (21.7%)			C (14.5%)				
	#4		V (38%)	I (47.4%)	I (44.7%)			K (47.7%)				K (47.5%)		D (100%)	Y (100%)												
	#5			I (91.4%)	I (91%)							K (93.1%)		D (100%)	Y (100%)												
	#6			I (17.8%)	I (23.6%)		Q (18.8%)	K (64.2%)				K (25.2%)		D (100%)	Y (100%)												
	#7							K (96.9%)					G (96.7%)	D (100%)	Y (100%)						I (93.9%)	Deletion (100%)				H (96.3%)	
dpi 30	#1	F (91.8%)	V (92.5%)	I (100%)	I (97.1%)							K (94.5%)		D (100%)	Y (100%)										C (40.6%)		
	#2			I (98.5%)	I (98.8%)							K (98.1%)		D (100%)	Y (100%)												
	#3				I (99%)					N (81.7%)		K (99.4%)		D (100%)	Y (99.9%)												
	#4					Deletion (32.9%)		K (95.3%)						D (100%)	Y (100%)									L (89.2%)			
	#5			I (10.9%)		Deletion (13.8%)		K (75%)				K (14.6%)		D (100%)	Y (100%)												
	#6		V (22.1%)	I (99%)	I (99.4%)							K (99.4%)		D (99.8%)	Y (100%)												
	#7				I (27.7%)			K (70%)				K (24.4%)		D (100%)	Y (100%)			F (24.7%)									

Data Availability Statement

All data needed to evaluate the conclusions in this manuscript have been included. The WGS data for the SARS-CoV-2 mutants analyzed in this study are available at the GISAID under the accession numbers: EPI_ISL_17375491, EPI_ISL_17375492, EPI_ISL_17375548, EPI_ISL_17375549, EPI_ISL_17375550, EPI_ISL_17375552, EPI_ISL_17375553, EPI_ISL_17375554, EPI_ISL_17375556, EPI_ISL_17375557, EPI_ISL_17375558, EPI_ISL_17375559, EPI_ISL_17375560, EPI_ISL_17375561, EPI_ISL_17375685, EPI_ISL_17375686, EPI_ISL_17375687, EPI_ISL_17375688, EPI_ISL_17375689, EPI_ISL_17375690, EPI_ISL_17375691, EPI_ISL_17375692, EPI_ISL_17375693, EPI_ISL_17375694, EPI_ISL_17375695, EPI_ISL_17375696. EPI_ISL_18065759. EPI_ISL_18065760.
